# Health-related quality-of-life evaluation in epidermolysis bullosa: a scoping review protocol

**DOI:** 10.1186/s13643-025-02918-9

**Published:** 2025-08-01

**Authors:** Greta Arias-Merino, Juan Benito-Lozano, Renata Linertová, Verónica Alonso-Ferreira, on behalf the BUR-EB Study Group

**Affiliations:** 1 Institute of Rare Diseases Research (IIER), Instituto de Salud Carlos III (ISCIII), 28029 Madrid, Spain; 2 Universidad Nacional de Educación a Distancia (UNED), 28015 Madrid, Spain; 3 Canary Islands Health Research Institute Foundation (FIISC), 38109 Santa Cruz de Tenerife, Spain; 4 Research Network On Chronicity, Primary Care, and Health Promotion (RICAPPS), Carlos III Health Institute (Instituto de Salud Carlos III), Madrid, Spain; 5 Spanish Network of Agencies for Health Technology Assessment for the National Health Service (RedETS), Madrid, Spain; 6 Centre for Biomedical Network Research On Rare Diseases (CIBERER), Instituto de Salud Carlos III, 28029 Madrid, Spain

**Keywords:** Questionnaires, Quality of life, Heath-related quality of life, Epidermolysis bullosa, Scoping review, Rare diseases

## Abstract

**Background:**

Epidermolysis bullosa (EB) is a group of rare congenital inherited skin diseases. Evaluation of health-related quality of life (HRQoL) is key to understanding the psychosocial and emotional impact on patients and their relatives or carers. The BUR-EB project aims at ascertaining the socio-economic burden of the disease, including its impact on costs and HRQoL. The aim of this scoping review is to identify which instruments or questionnaires are being used to evaluate the HRQoL of patients with EB and their relatives and carers.

**Methods:**

A scoping review will be conducted to ascertain the HRQoL of EB patients, their family circle and caregivers over the past 12 years. The wide-ranging review question is as follows: “Which measurement tools are available for evaluation of HRQoL in EB?” A search will be made in the MEDLINE, WOS and Scopus databases, and the Joanna Briggs Institute methodology will be applied. The data and findings will be shown in tables and in narrative form, in line with the Preferred Reporting Items for Systematic reviews and Meta-Analyses extension for Scoping Reviews (PRISMA-ScR).

**Discussion:**

This scoping review will provide an overview of evidence on the use of HRQoL instruments, both in patients with EB and in relatives and carers. The results of this scoping review will offer a guidance to the researchers to measure the health and well-being of EB patients.

**Supplementary Information:**

The online version contains supplementary material available at 10.1186/s13643-025-02918-9.

## Background

Epidermolysis bullosa (EB) is a group of rare congenital inherited skin diseases, characterised by fragile skin lesion and blister formation, inducible by the slightest trauma [1, 2]. These conditions can vary in their severity, ranging from mild forms to debilitating and potentially mortal cases [3]. There are four major types of EB: EB simplex (EBS), junctional EB (JEB), dystrophic EB (DEB) and Kindler EB (KEB) [4]. All types and subtypes of EB are rare, and the prevalence of EB is estimated at 4–54:1,000,000 [5]. This rare disease (RD) has no cure and poses an enormous disease and social and financial burden for the affected persons and their families.

Evaluation of health-related quality of life (HRQoL) in patients with EB is a fundamental aspect for understanding the psychosocial and emotional impact that this disease has on the lives of those affected and for informing clinical decision-making and the development of appropriate interventions [6]. The chronic, painful and debilitating nature of EB can generate a series of challenges in patients’ daily lives. Hence, evaluation of quality of life in this context is not confined to measuring physical aspects but also takes into account the emotional, social and psychological aspects. Similarly, it is not limited to patients themselves, since it is important to evaluate the quality of life of their relatives and carers, who are also affected.

Evaluation of HRQoL in EB patients is useful, not merely for guiding medical care but also for generating awareness about the importance of research and the development of specific therapies. By understanding the profound impact of EB on patients’ lives, researchers can direct their efforts towards solutions which address both the physical and the emotional and psychosocial aspects of the disease.

Furthermore, regular, integrated evaluation of HRQoL in health systems can be useful for measuring the efficacy of medical and therapeutic interventions over time. Changes in quality-of-life scores may reflect patients’ response to treatment, thereby allowing health professionals to tailor and personalise care as required.

In 2011, the European BURQOL-RD project analysed the socio-economic and HRQoL burden of EB, between other RDs. Currently, the “Changes in the Socio-economic Burden of Epidermolysis Bullosa in Europe” BUR-EB project funded by the European Union (EU) seeks to update information obtained more than 10 years ago, estimate the socio-economic burden of EB in 7 EU countries and compare this against data obtained in the BURQOL-RD study. The BURQOL-RD results, gathered more than 10 years ago, revealed a considerable financial burden of EB for affected families and society in general [7]. The current aim is to analyse possible changes due to the introduction of new treatments, public policies or care models.

There are EB-specific HRQoL evaluation instruments which seek to capture the impact of disease in multiple dimensions. Based on an initial exploratory search, it has been possible to establish that some of the most used are the “Quality of Life Evaluation in Epidermolysis Bullosa (QoLEB)” [8] and “Epidermolysis Bullosa Burden of Disease (EB-BoD)” questionnaires [9]. Some more general instruments for dermatological diseases are also used, such as the “Family Dermatology Life Quality Index (FDLQI)” [10] and the “Children’s Dermatology Life Quality Index (CDLQI)” [11], which is applied to children with EB.

Insofar as published scientific literature is concerned, there are no specific reviews of the study topic. There are, however, some studies that do measure the quality-of-life impact of EB, whether in respect of the family and health professionals [6, 12] or bullous diseases [13], but do not address instruments and questionnaires specifically. There are also others on the symptomatology, disease burden and socio-economic burden of EB [14], as well as systematic reviews on the use of instruments in patients with congenital abnormalities [14], in dermatological disorders (in which a single type of EB questionnaire is included) [15], but not specifically for patients with EB. Due to the fact that in the BUR-EB European project a questionnaire is to be drawn up to evaluate HRQoL in EB patients and their relatives, it is relevant to ascertain which instruments are being used.

The aim of this scoping review will therefore be to identify the instruments used to evaluate HRQoL and their applicability to EB and those directly and indirectly affected by it.

### Review questions

The main question for the proposed scoping review is as follows: “Which measurement tools are available for evaluation of HRQoL in EB?”. The following subquestions are also posed to guide the study:

- Are the HRQoL instruments found EB specific?
- Are there HRQoL questionnaires that are validated and adapted by age group?
- Which HRQoL questionnaires are being used for EB patients’ carers and/or relatives?

### Eligibility criteria

#### Participants

This scoping review will consider all types of studies in which some HRQoL-specific or generic questionnaire has been used. It will also include instruments that have been applied to EB patients or to their relatives or carers, with the aim of assessing HRQoL. There will be no restrictions by type of EB, age, sex, culture or ethnicity.

#### Concept

All the instruments that measure HRQoL, whether general or EB specific, will be mapped. The scoping review would also be able to identify psychometric characteristics, as well as the specificity of questionnaires according to their respective context (validated and/or adapted questionnaires).

An effort has been made to be broad based in the concept of quality of life (QoL), including terms such as"Quality of Life/"Health Related Quality of life". In addition, concepts have been included which cover other QoL-related aspects, ranging from psychological and well-being aspects ("Psychological Well-Being"/"well-being") to those related with patients’ health (“illness burden”) and the impact on their relatives and carers (“family burden"/"Caregiver Burden").

The RD of interest for this scoping review is EB. All studies that address any of the types of EB (EBS, JEB, DEB and KEB) will be included.

#### Context

This review will consider studies covering any geographical location, region, city or country. Moreover, studies will not be limited by reference to medical care, primary care or hospital setting.

#### Type of sources

This scoping review will consider all papers that describe questionnaires, surveys or instruments, both generic and specific, which measure the HRQoL of EB patients, their relatives and carers. The questionnaires may be validated or adapted. Original research studies and conceptual/theoretical papers will be considered. Review-type studies, systematic reviews, meta-analyses and letters to the editor will be excluded.

## Methods

The proposed scoping review will be conducted in accordance with the Joanna Briggs Institute (JBI) methodology for scoping reviews [16–18] and as per the Preferred Reporting Items for Systematic reviews and Meta-Analyses extension for Scoping Reviews (PRISMA-ScR) [19].

### Search strategy

A limited pilot search will be conducted in MEDLINE (PubMed), in order to identify papers on the topic and define the key words (Appendix I: Search strategy).

The words of the text contained in the titles and abstracts of relevant papers and the index terms used to describe papers were used to develop a comprehensive search strategy. The final search strategy will seek to locate papers published in MEDLINE (PubMed), Scopus and Web of Science (WOS). The search strategy, including all the key words identified and index terms, will be tailored to each data source included. Date of publication will be limited to the period from 23 February 2011 to 01 October 2023, and the search will be restricted to the English language and journal articles.

### Source of evidence selection

All papers identified will be collected and uploaded to the Zotero reference manager, and duplicates will be eliminated after the search. Two independent reviewers will examine the titles and abstracts for selection purposes in line with pre-defined inclusion criteria. The full text of all papers selected in accordance with the inclusion criteria will undergo a detailed peer review. The reasons for exclusion of full-text studies which do not fulfil the inclusion criteria will be noted and reported in the scoping review. Any differences of opinion will be settled by consensus or by decision of a third reviewer.

The results of the search and the study inclusion process will be fully reported in the final scoping review and will be shown in a PRISMA flow diagram [16, 19, 20].

### Data extraction

Data will be extracted from the papers included in the scoping review by two independent reviewers, using a data extraction tool purpose developed by the reviewers (Appendix II). The data extracted will include specific details on the following: (1) General information about the studies, (2) the characteristics of the questionnaires, (3) the characteristics of the study participants, (4) the validity and reliability of the questionnaires, and (5) the instrument’s domains. The draft data extraction tool will be amended and revised as required during the process of extracting data from each of the studies included, and all the amendments will be described in the complete report of the scoping review. Papers’ authors will be contacted to request any missing measurement tools, where necessary.

### Data analysis and presentation

After the extraction of data, the pertinent information will be shown in tables and figures, in a way that is in consonance with the designated aim of this scoping review. A narrative summary will accompany the tabulated results and/or graphs. This will describe how the results relate to the aim of the review and the main research question, as well as the subquestions.

The descriptions of the studies included will be provided in tables. Details of bibliometric indicators and principal conclusions will be listed according to the table shown in Appendix II.

## Discussion

HRQoL-related research has witnessed significant development across the period 2000–2019, in terms of both generic and specific HRQoL instruments [21]. That said, however, this development is targeted at the most prevalent diseases [22]. Insofar as RDs are concerned, there is the previous example of the 2011 European BURQOL-RD project which analysed the socio-economic and HRQoL burden of 10 RDs, including EB [23]. In that study, the validated EQ-5D questionnaire was used in seven European countries [24]. More than a decade after that intervention, the idea arose of using this scoping review to ascertain if new instruments for measuring HRQoL in EB have been developed, validated or adapted and if there are specific instruments by age group, for patients, relatives and carers, taking into account the context or whether or not they are integrated into healthcare. The results of this scoping review will provide key data which will enable health professionals and social workers alike to better understand an individual’s opinions about his/her health and well-being. To this end, a broad-based scoping review of key words and index terms will be conducted. Furthermore, a wide spectrum of published literature will be covered. This review provides a transparent, reproducible search strategy in accordance with JBI methodology updated for scoping reviews [18].

A limitation of this review is the inclusion of literature published only in the English language, which may produce a language bias by excluding relevant papers published in other languages. As with most literature reviews, there is a potential risk of publication bias, as studies reporting significant results are more likely to be published. It is also possible to expect that the studies found could have a small sample size, as the EB is a RD. In order to mitigate these limitations, we will adhere to the rigorous research methods outlined in the methodology section of this protocol, using the three most sensitive databases in the clinical field and strive to present the complex aspects of this research theme with the greatest possible accuracy and comprehensiveness.

This scoping review will provide an overview of evidence on the use of HRQoL instruments, both in EB patients and in their relatives and carers.

## Supplementary Information

Appendix 1. Search strategy.

Appendix 2. Data-extraction instrument.

Supplementary Material 3. Preferred Reporting Items for Systematic reviews and Meta-Analyses extension for Scoping Reviews (PRISMA-ScR) Checklist.

## Data Availability

Not applicable.

## Abbreviations

CDLQI: Children’s Dermatology Life Quality Index
DEB: Dystrophic epidermolysis bullosa
EB: Epidermolysis bullosa
EBS: Epidermolysis bullosa simplex
EB-BoD: Epidermolysis bullosa burden of disease
EU: European Union
FDLQI: Family Dermatology Life Quality Index
JEB: Junctional epidermolysis bullosa
KEB: Kindler epidermolysis bullosa
HRQoL: Evaluation of health-related quality of life
PRISMA-ScR: Preferred Reporting Items for Systematic Reviews and Meta-Analyses extension for Scoping Reviews
RD: Rare Diseases
QoLEB: Quality of Life Evaluation in Epidermolysis Bullosa
