# Supplementary material for: Health-related quality-of-life evaluation in epidermolysis bullosa: a scoping review protocol
Source: Syst Rev. 2025 Aug 1;14:159. doi: 10.1186/s13643-025-02918-9 (PMC12317451; doi:10.1186/s13643-025-02918-9)
Supplement: Supplementary file 1 — Appendix 1. Search strategy. [file 13643_2025_2918_MOESM1_ESM.pdf]

Appendix I: Search strategy

MEDLINE (via PUBMED platform)

(("Epidermolysis Bullosa"[Mesh] OR "epidermolysis bullosa "[Title/Abstract] OR "Poikiloderma of Kindler" OR "poikiloderma" [Title/Abstract] OR ("poikiloderma" AND "kindler") OR "kindler-syndrome" [Title/Abstract])

AND ("Quality of Life"[Mesh] OR "Quality of life"[Title/Abstract] OR "Health Related Quality of life" [Title/Abstract] OR "Psychological Well-Being"[Mesh] OR "well-being"[Title/Abstract] OR "wellbeing"[Title/Abstract] OR "Caregiver Burden"[Title/Abstract] OR "Caregiver Burden"[Mesh] OR "Cost of Illness"[Mesh] OR "burden of illness" [Title/Abstract] OR "illness burden" [Title/Abstract] OR (("family"[Title/Abstract]) AND ("burden"[Title/Abstract])) OR "family burden")

AND ("Surveys and Questionnaires"[Mesh] OR "Surveys and Questionnaires"[Title/Abstract] OR "Personal Construct Theory"[Mesh] OR "questionnaire" OR "survey" OR "interview" OR "instrument")) Filters: English, from 2011/2/23 - 2023/10/1
