# Supplementary material for: Health-related quality-of-life evaluation in epidermolysis bullosa: a scoping review protocol
Source: Syst Rev. 2025 Aug 1;14:159. doi: 10.1186/s13643-025-02918-9 (PMC12317451; doi:10.1186/s13643-025-02918-9)
Supplement: Supplementary file 2 — Appendix 2. Data-extraction instrument. [file 13643_2025_2918_MOESM2_ESM.pdf]

Appendix II: Data-extraction instrument

**General information**<br><br>(Study/author /year)	**Study characteristics**<br><br>(1) Type of study<br>(2) Instrument name used in the study<br>(3) Number of items<br>(4) General or specific	**Characteristics of study participants**<br><br>(1) Number/population<br>(2) Type of EB<br>(3) Age<br>(4) Sex<br>(5) Patient /carer/family<br>(6) Context<br>(7) Country and city	**Psychometric characteristics**<br><br>(1) Cultural or linguistic adaptation<br>(2) Reliability<br>(3) Validity	**Pertinent domain of instrument**<br><br>(1) EB-specific quality of life<br>(2) General quality of life<br>(3) Dermatological quality of life<br>(4) Quality of life associated with symptoms<br>(5) Quality of life and socio-economic cost<br>(6) Other
